# Smoking habits and the influence of war on cigarette and shisha smoking in Syria

**DOI:** 10.1371/journal.pone.0256829

**Published:** 2021-09-02

**Authors:** Ameer Kakaje, Mohammad Marwan Alhalabi, Ayham Alyousbashi, Ayham Ghareeb, Loura Hamid, Ala’a B. Al-Tammemi

**Affiliations:** 1 Faculty of Medicine, Damascus University, Damascus, Syria; 2 University Hospital Geelong, Barwon Health, Victoria, Australia; 3 Faculty of Medicine, Department of Family and Occupational Medicine, University of Debrecen, Debrecen, Hungary; 4 Doctoral School of Health Sciences, University of Debrecen, Debrecen, Hungary; University of South Carolina College of Pharmacy, UNITED STATES

## Abstract

Tobacco smoking might be impacted by various influences, including psychological, socio-cultural, and economic factors. A community-based cross-sectional survey was conducted in Syrian Arab Republic from March to April 2019 using a web-based questionnaire. The survey aimed at assessing tobacco use (shisha and cigarettes) as well as examining the association between current tobacco use and various sociodemographic and war-related factors. The sample comprised 978 participants (251 males: 727 females) and had a mean age of 24.7 years (SD: 7.60). Most participants were single (n = 825, 84.4%), reside in Damascus and Rif-Dimashq (n = 579, 59.2%), and had a college/university education (n = 911, 93.1%). Concerning smoking, a total of 371 participants (37.9%) were identified to be current tobacco smokers, of whom 211, 84, 76 were exclusive shisha smokers, exclusive cigarette smokers, and dual smokers, respectively. The prevalence of cigarette smoking (exclusive and dual) among males and females was found to be 34.7%, and 10.0%, respectively. On the other hand, the prevalence of shisha smoking (exclusive and dual) among males and females was around 34.3% and 27.6%, respectively. Additionally, various factors have predicted a higher likelihood of cigarette smoking including male gender (AOR = 4.152; 95% CI: 2.842–6.064; p<0.001), and losing someone due to the war (AOR = 1.487; 95% CI: 1.028–2.151; p = 0.035), while unemployed individuals were found to have lower odds of being cigarette smokers (AOR = 0.634; 95% CI: 0.429–0.937; p = 0.022). Concerning shisha smoking, married (AOR = 0.622; 95% CI: 0.402–0.963; p = 0.033), and unemployed individuals (AOR = 0.679; 95% CI: 0.503–0.916; p = 0.011) were found to have lower odds of shisha smoking. Amid the tobacco epidemic in the region, rates of tobacco use in Syria are still worrying. The Syrian armed conflicts may possess a double-edged effect on smoking, and tobacco users who adopt smoking to cope with various stressors should be targeted with well-structured health education, along with appropriate psychological services.

## 1. Background

Tobacco smoking is a major risk factor for various preventable medical conditions with an estimation of eight million deaths that are caused by tobacco-related diseases every year [1]. Smoking can cause substantial economic losses, and it can contribute to poverty, as it is an addictive habit that forces individuals to prioritize buying tobacco over their basic needs [1]. Globally, around 80% of smokers reside in low- and middle-income countries, where most tobacco-induced morbidity and mortality also occur [1–3] In addition to active smoking, passive smoking can also be harmful, as it was proven to cause serious cardiovascular and respiratory diseases similar to active smoking. Tobacco products can be utilized whether in combustible forms (cigarettes, shisha, cigar, pipe.. etc) or smokeless forms (chewed, dry snuff, moist snuff, snus) [4].

Shisha, also known as hookah, waterpipe, narghile, or Hubble bubble, is a popular form of smoking that has serious harmful effects on health. Shisha smoking is becoming an epidemic and has been spreading since the 1990s, especially after the introduction of flavored and aromatic shisha tobacco (known as Ma’assel) [5]. In addition to cigarette smoking, shisha is very popular in countries within the Middle East and North Africa (MENA) such as Egypt, Jordan, Lebanon, Saudi Arabia, and Syria [6,7]. Shisha smoking is considered a pleasurable social experience that contributes to the growing popularity of this smoking method, while cigarette smoking is seen as a personal addiction [8]. Shisha is mostly smoked indoors in cafés, shisha bars, or at home, and this may harm many non-smokers who are exposed to passive smoking. Besides, benzene and 3-hydroxypropylmercaptruic acid were found in the urine of passive smokers, while nitrosamine from tobacco and acrolein were found in children who lived with shisha smokers [9–11].

The Syrian Arab Republic, most popularly known as Syria, has been in continuous armed conflicts and political unrest since 2011 which forced millions of Syrians to whether being internally displaced from their usual place of residence or to move out of the country as refugees to find peaceful life elsewhere. The war crisis has severely impacted various life domains in Syria and has negatively afflicted the economy, education, and social life of Syrians [12]. Moreover, mental disorders such as posttraumatic stress disorder (PTSD), depression, and anxiety were a remark of the Syrian war and the associated deterioration of living conditions [13]. The armed conflict and its related psychological pressure may have serious impacts and could be associated with risky behaviors such as initiation of smoking, increased smoking, or even substance abuse (psychoactive drugs, alcohol). It was reported that various environmental and psychosocial stressors caused by man-made disasters (e.g., armed conflicts, interpersonal victimization) or natural disasters could have serious effects on smoking behavior, resulting in a high rate of tobacco use [14]. Notably, the Eastern Mediterranean Region (EMR) is characterized by high rates of tobacco use with an estimation of 3.0%, 6.1%, and 3.8% of exclusive cigarette, exclusive shisha, and dual-use, respectively [14,15].

Syria has a unique environment and practices such as the popularity of shisha smoking as a part of daily socialization [16,17], and this negatively impacts people’s health and increase their susceptibility to many illnesses such as allergic rhinitis [18], laryngopharyngeal reflux [19], cardiovascular diseases, pulmonary diseases, and cancers [20]. Notably, smoking among young people in Syria is not uncommon as youths and young adults perceive it as a pleasurable experience, in addition to peer pressure and the effect of parental smoking [16]. For instance, current smoking amongst high school students in Syria had reached a prevalence of 15.9% among males and 6.6% among females in 2000 [16]. In 2015, a study has reported a prevalence of current smoking among male and female university students in Syria of 39.8%, and 5.5%, respectively [12].

Taking into consideration many factors on the personal, local, national, and regional levels including, the armed conflicts and the accompanying political unrest that resulted in significant socio-economic and psychological impacts on the Syrian population over the past decade, the dynamicity of human behaviors, high smoking rates in the EMR, and the effect of stressful events on risky and unhealthy behaviors, our present study aimed to assess cigarette and shisha smoking in a community-based sample from Syria, as well as to examine the association of current tobacco smoking with various socioeconomic and war-related predictors. Our specific research questions were, (i) what is the prevalence of current tobacco smoking (cigarette, shisha) in the Syrian community? (ii) What sociodemographic factors may predict current tobacco use? and (iii) How did the war crisis impact current tobacco use?

## 2. Methods and materials

### 2.1 Study design and sampling

This was an online questionnaire-based cross-sectional survey that was conducted in Syria from March 2019 to April 2019. The questionnaire was created using Google Form^®^ and was delivered in modern standard Arabic, the official national language in Syria. The survey questionnaire was disseminated to Syrian participants on Facebook^®^ groups. The high level of accessibility to this social media platform by most people in Syria has helped us to eliminate the geographical boundaries aiming to reach participants from different Syrian governorates. For any individual to be eligible for enrolment in our study, all the following criteria should have been met and were explained to the community in the cover letter of our survey, including (i) Arabic-speaking individual of age 16 years or above (ii) lived inside Syria in the past year, (iii) responding to gender and current tobacco use items, and (iv) providing informed consent by ticking a box that declares the participant’s complete understandability about the research study and its objectives. Participation in our study was voluntary without providing any incentives or rewards. A convenience sampling strategy was employed to recruit participants.

### 2.2 Survey instrument and related measures

Our survey questionnaire comprised three sections (S1 File). The first section collected basic sociodemographic data such as gender (male; female), age (in years), marital status (single; married), educational level (up to high school, college/university education), the governorate of current residence (Damascus and Rif Dimashq; Daraa; Al-Raqqah; As-Suwayda; Deir Ezzor; Latakia; Al-Hasakah; Hama; Idleb; Tartous; Homs; Aleppo), employment status (employed; unemployed), and self-rated socioeconomic status (SES) (lower, middle, upper). Regarding SES, it cannot be reliably assessed in Syria as asking for monthly income is not welcomed and due to the difference between the living expenses between Syria and other countries where SES questionnaires were validated [13]. Also in the first section, the participants were asked to report if they had any diagnosed chronic medical condition (medically free; pulmonary conditions; other medical conditions like hypertension, Diabetes, gastrointestinal. etc).

The second section included several items related to the tobacco use profile of the participants. The participants were asked to self-report their past 30 days of tobacco use concerning cigarettes and shisha smoking (were classified into currently non-smoker, currently smokers). Further, the participant was asked to report specifically which smoking methods were used in the past 30 days (cigarette, shisha, dual-use). Besides, participants who self-reported to be current cigarette or shisha smokers were requested to answer more items related to smoking patterns (two items for cigarette smokers and three items for shisha smokers), including approximate duration of cigarette smoking (in years), number of cigarette packs smoked per day, the approximate duration of a single shisha session (in hours), frequency of shisha sessions per week, and the preferred time for smoking shisha (unspecified, morning, during social gathering).

In the third section of our questionnaire, the participants were requested to answer three items regarding the impacts of the Syrian war crisis on personal life, including changing the usual place of residence due to the war (yes; no), losing someone due to the war (yes; no), and lastly if being distressed from war-induced noises (yes; no).

For the purpose of assessing the face and content validity, phrasing, and clarity of the questionnaire, it was assessed by two academics followed by a pilot-testing on 50 participants. Minor linguistic modifications were applied to the questionnaire based on the feedback from the piloting phase. The pilot responses were not included in our analysis. For sample size estimation, we used Open Source Epidemiologic Statistics for Public Health (OpenEpi, Atlanta, GA) software version 3.01 [21]. A sample size of at least 385 participants was required for our study with a 95% confidence level, 5% margin of error, and 50% response distribution.

### 2.3 Data analysis

Data were extracted from Google Form^®^ as an Excel sheet for quality check, data cleaning, and coding. Then, the Excel sheet was exported into Statistical Package for Social Sciences version 26.0 (SPSS Inc., Chicago, IL, USA) for further statistical analyses. Descriptive statistics were used in which continuous variables were described as mean and standard deviation (SD), while categorical variables were reported as frequency counts and percentages. Pearson’s Chi-square test was used to detect significant differences between smoking groups according to sociodemographic and war-related factors. Additionally, three multivariable logistic regression models for *cigarette smoking (model 1)*, *shisha smoking (model 2)*, as well as *cigarette and/or shisha smoking—overall tobacco use (model 3)* were created to examine the association between tobacco use status and various sociodemographic and war-related factors. Explanatory variables that had a p-value < 0.2 in the univariable model were considered as candidate variables for the multivariable regression [22]. Both unadjusted odds ratio (OR) and adjusted odds ratio (AOR) with their 95% confidence intervals (CI) were reported. A p-value < 0.05 was implemented for statistical significance.

### 2.4 Ethical considerations

The ethical permission for conducting our study was obtained from the ethical committee—Faculty of Medicine at Damascus University in Syria. All methods were carried out following the institutional and national guidelines and conforming to the ethical standards of the declaration of Helsinki. All participants were informed about the study objectives and written informed consent was obtained as a prerequisite before administering the questionnaire.

## 3. Results

### 3.1 Sociodemographic characteristics

Our sample had 978 participants with a mean age of 24.7 years (SD: 7.6), and the majority of participants (n = 835, 85.4%) fell into the age group of 18–30 years. Most of the participants were females (n = 727, 74.3%), single (n = 825, 84.4%), reside in Damascus and Rif-Dimashq (n = 579, 59.2%), had a college/university education (n = 911, 93.1%), were currently non-smokers (n = 607, 62.0%), with middle SES (n = 712, 72.8%), were unemployed (n = 620, 63.4%), and were medically free (n = 551, 56.3%). Table 1 shows more details about sociodemographic characteristics of the participants.

**Table 1:** Socio-demographic characteristics of the respondents (n = 978).

Characteristic	Frequency Count (n)^┼^	Percentage (%)^┼^
**Age (Mean, SD) =** 24.7 years (SD: 7.6)		
**Gender**		
Male	251	25.7
Female	727	74.3
**Marital Status**		
Single (including divorced and widowed)	825	84.4
Married	144	14.7
**Governorate of Residence**		
Damascus & Rif-Dimashq	579	59.2
Daraa	3	0.31
Al-Raqqah	1	0.10
As-Suwayda	21	2.15
Deir Elzzor	1	0.10
Latakia	65	6.65
Al-Hasakah	3	0.31
Hama	36	3.7
Idleb	4	0.41
Tartous	29	2.98
Homs	92	9.41
Aleppo	62	6.34
**Educational level**		
Up to high School	62	6.3
College/University Education	911	93.1
**Current Tobacco Smoking**		
Non-Smoker	607	62.0
Exclusive Shisha	211	21.6
Exclusive Cigarettes	84	8.6
Cigarettes and shisha (dual)	76	7.8
**Socio-Economic Status (SES) Level**		
Lower	244	25.0
Middle	712	72.8
Upper	22	2.2
**Employment Status**		
Unemployed	620	63.4
Employed	351	35.9
**Chronic medical conditions**		
No	549	56.1
Pulmonary conditions, Asthma	67	6.9
Other medical conditions	230	23.5

^**┼**^ Some counts and percentages do not add up to exact 978 and 100%, respectively, due to some missing values.

### 3.2 Smoking profile of the participants

**Fig 1:**
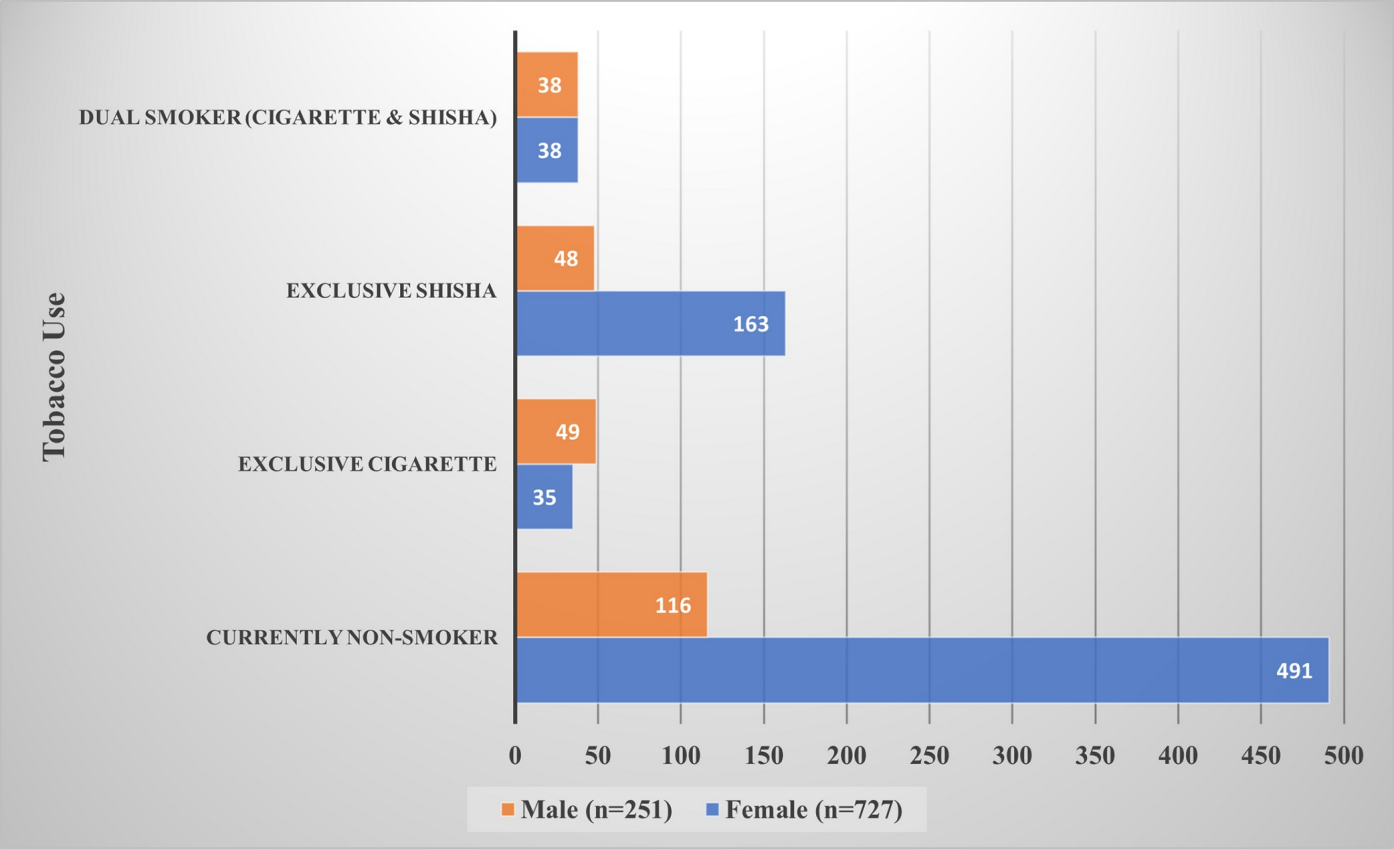
Current tobacco smoking among females and males (frequency counts).

**Fig 2:**
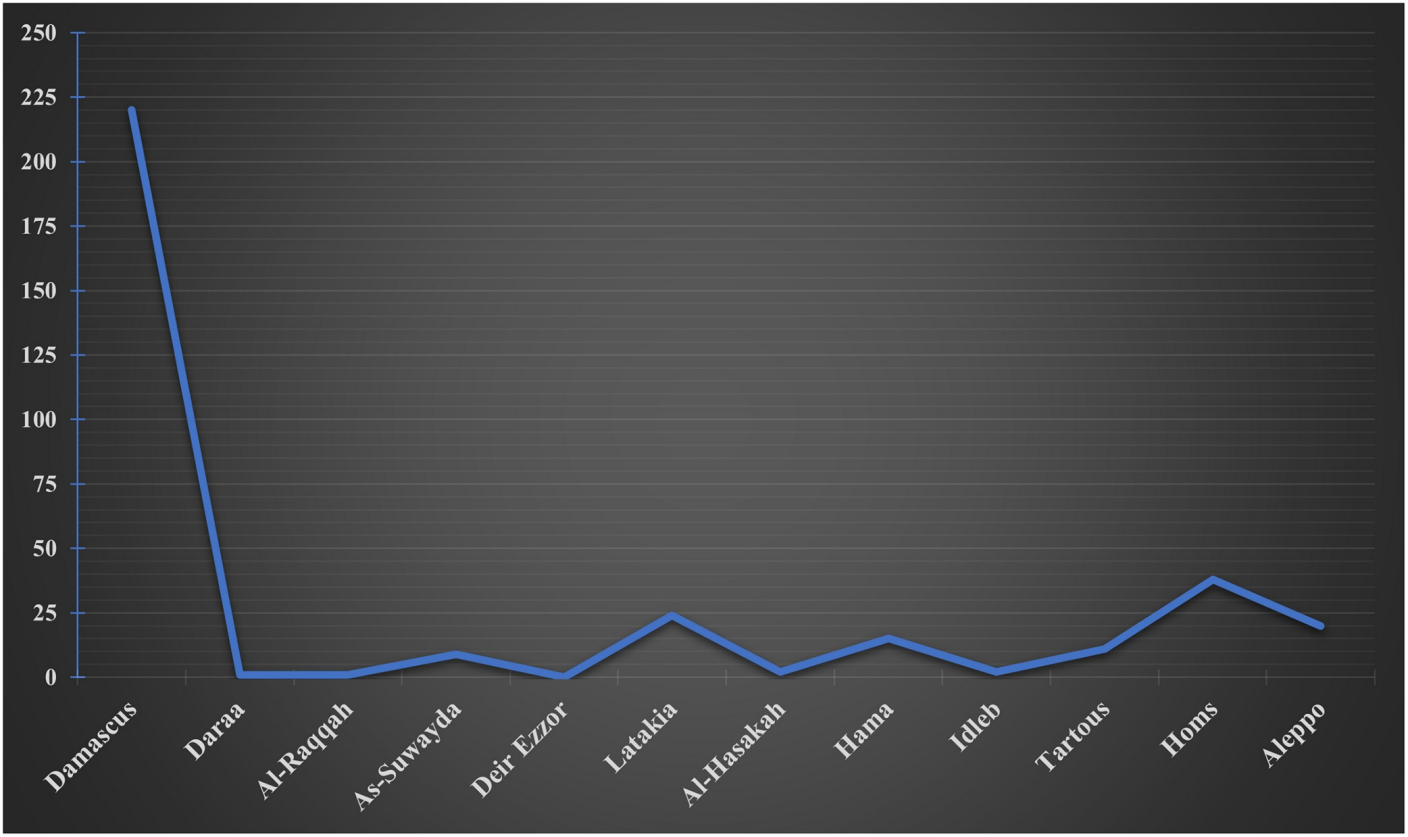
Current tobacco smoking among the participants based on the governorate of residence in Syria (frequency counts).

Concerning tobacco smoking, a total of 371 participants were found to be current tobacco users, of whom 211, 84, 76 were exclusive shisha smokers, exclusive cigarette smokers, and dual smokers, respectively. Therefore, cigarette smokers (exclusive and dual) made up to 160 participants, while shisha smokers (exclusive and dual) made up to 287 participants. The prevalence of cigarette smoking (exclusive and dual) among males and females was found to be 34.7%, and 10.0%, respectively (p<0.001). On the other hand, the prevalence of shisha smoking (exclusive and dual) among males and females was around 34.3% and 27.6%, respectively (p = 0.047). Fig 1 shows that among current tobacco smokers, most exclusive shisha smokers were females (n = 163), however, gender distribution was closely similar regarding dual smoking and exclusive cigarette smoking. Concerning geographical distribution, Fig 2 illustrates that of the overall current tobacco smokers (n = 371), the majority were residing in Damascus and Rif-Dimashq (n = 220), followed by those from Homs (n = 38), Latakia (n = 24), and Aleppo (n = 20). Moreover, Table 2 provides a comparison between different smoking groups according to the socio-demographic characteristics of the participants.

**Table 2:** Comparing smoking status of participants according to socio-demographic factors.

	Cigarette Smoking			Shisha Smoking			Current Tobacco Smoking (Cigarette and/or Shisha Smoking)		
	No	Yes (n = 160)	Chi-square p-value	No	Yes (n = 287)	Chi-square p-value	No (n = 607)	Yes (n = 371)	Chi-square p-value
**Gender**									
**Male**	164	87	**<0.001**	165	86	**0.047**	116	135	**<0.001**
**Female**	654	73		526	201		491	236	
**Marital Status**									
**Single**	698	127	0.066	572	253	**0.040**	514	311	0.556
**Married**	113	31		112	32		86	58	
**Educational Level**									
**Up to High School**	50	12	0.507	50	12	0.078	40	22	0.695
**College/University**	764	147		639	272		565	346	
**Socioeconomic Status (SES)**									
**Lower**	197	47	0.114	181	63	0.374	147	97	0.600
**Middle**	605	107		495	217		448	264	
**Upper**	16	6		15	7		12	10	
**Age [Mean (SD)]** ^$^									
	24.3 (7.4)	26.5 (8.4)	**<0.001**	24.9 (8.3)	24.2 (5.5)	0.202	24.4 (9.9)	25.2 (7.0)	0.116
**Employment status**									
**Unemployed**	545	75	**<0.001**	456	164	**0.008**	412	208	**<0.001**
**Employed**	267	84		230	121		190	161	
**Medical condition**									
**No**	457	92	0.209	382	167	0.383	332	217	0.206
**Pulmonary, asthma and allergy**	60	7		52	15		48	19	
**Other medical conditions**	185	45		164	66		141	89	

Staistically significant p values at p<0.05 are marked in bold.

^$^One-Way ANOVA.

Among cigarette smokers, the average duration of smoking was estimated to be around 5.70 years (SD: 5.02), with an average number of packs per day of 1.14 (SD: 0.50). On the other hand, shisha smokers were found to have a mean duration per single session of 1.06 hours (SD: 0.58), and a mean of 3.07 shisha sessions per week (SD:3.55). Regarding the preferred time for shisha smoking among its users, most shisha smokers reported that they usually preferred it in social gatherings and events (208/287), while others (70/287) preferred it in the morning times. However, a small proportion of shisha smokers (9/287) had no specific preference.

### 3.3 War-related impacts

Regarding war-related impacts on personal life, around 31.5% of participants (n = 308) were forced to change their usual residence place due to the war crisis, 42.2% (n = 413) had lost someone due to the war, and approximately 63.1% of the participants (n = 617) were distressed by war-induced noises. More details are provided in Table 3.

**Table 3:** War -related impacts among the participants according to current smoking status.

	Cigarette Smoking			Shisha Smoking			Current Tobacco Smoking (Cigarette and/or Shisha Smoking)		
	No	Yes (n = 160)	Chi-square p-value	No	Yes (n = 287)	Chi-square p-value	No (n = 607)	Yes (n = 371)	Chi-square p-value
**Losing someone close due to war**									
**No**	480	73	**0.002**	402	151	0.083	366	187	**0.002**
**Yes**	327	86		279	134		232	181	
**Being distressed from war noises**									
**No**	281	71	**0.014**	238	114	0.154	203	149	**0.040**
**Yes**	530	87		444	173		397	220	
**Changing place of living due to war**									
**No**	555	101	0.224	452	204	0.128	402	254	0.545
**Yes**	251	57		227	81		195	113	

^**┼**^ Some counts do not add up due to some missing values.

### 3.4 Findings of multivariable logistic regression analysis

As previously described in the data analysis section, three multivariable logistic regression models were created to assess the association between various sociodemographic and war-related factors with the tobacco use status concerning cigarette smoking (model 1), shisha smoking (model 2), and cigarette and/or shisha smoking (overall tobacco use) (model 3).

*Model 1* has revealed a statistically significant association between current cigarette smoking and male gender (AOR = 4.152; 95% CI: 2.842–6.064; p<0.001), unemployment (AOR = 0.634; 95% CI: 0.429–0.937; p = 0.022) and losing someone due to the war (AOR = 1.487; 95% CI: 1.028–2.151; p = 0.035). *Model 2* revealed a statistically significant relationship between current shisha smoking and married status (AOR = 0.622; 95% CI: 0.402–0.963; p = 0.033), and unemployment (AOR = 0.679; 95% CI: 0.503–0.916; p = 0.011). In *model 3*, we found statistically significant association between current tobacco smoking (cigarette and/or shisha) and male gender (AOR = 2.152; 95% CI: 1.583–2.927; p<0.001), unemployment (AOR = 0.690; 95% CI: 0.513–0.927; p = 0.014) and losing someone due to the war (AOR = 1.383; 95% CI: 1.053–1.817; p = 0.020). Table 4 represents the regression analyses results.

**Table 4:** Multivariable logistic regression analysis for the association between type of tobacco smoking and various predictors^┼^.

Predictors Category	Subcategory	Model 1: Current Cigarette Smoking				Model 2: Current Shisha Smoking				Model 3: Current Tobacco Smoking (Cigarette and/or Shisha Smoking)			
		Crude OR (95% CI)	*P value*	AOR (95% CI)	*P value*	Crude OR (95% CI)	*P value*	AOR (95% CI)	*P value*	Crude OR (95% CI)	*P value*	AOR (95% CI)	*P value*
**Age** ^$^	-	1.032 (1.012–1.052)	<0.001	1.022 (0.996–1.049)	0.094	-	-	-	-	1.013 (0.997–1.031)	0.118	1.003 (0.985–1.022)	0.718
**Gender**	Female	Reference				Reference				Reference			
	Male	4.753 (3.332–6.779)	<0.001	**4.152 (2.842–6.064)**	**<0.001**	1.364 (1.003–1.854)	0.048	1.275 (0.917–1.771)	0.149	2.406 (1.796–3.224)	<0.001	**2.152 (1.583–2.927)**	**<0.001**
**Marital status**	Single	Reference				Reference				-	-	-	-
	Married	0.663 (0.427–1.030)	0.067	1.166 (0.667–2.037)	0.590	0.646 (0.425–0.983)	0.041	**0.622 (0.402–0.963)**	**0.033**	-	-	-	-
**Socio-Economic Status (SES)**	Low	Reference				-	-	-	-	-	-	-	-
	Middle	0.741 (0.508–1.083)	0.121	0.949 (0.627–1.437)	0.805	-	-	-	-	-	-	-	-
	Upper	1.572 (0.584–4.233)	0.371	1.985 (0.669–5.887)	0.216	-	-	-	-	-	-	-	-
**Educational Level**	Up to high School	-	-	-	-	Reference				-	-	-	-
	College/University	-	-	-	-	1.774 (0.930–3.383)	0.082	1.346 (0.694–2.613)	0.380	-	-	-	-
**Employment status**	Employed	Reference				Reference				Reference			
	Unemployed	0.437 (0.310-.617)	<0.001	**0.634 (0.429–0.937)**	**0.022**	0.684 (0.515–0.908)	0.009	**0.679 (0.503–0.916)**	**0.011**	0.596 (0.456–0.779)	<0.001	**0.690 (0.513–0.927)**	**0.014**
**Losing Soemone close due to War**	No	Reference				Reference				Reference			
	Yes	1.729 (1.228–2.435)	0.002	**1.487 (1.028–2.151)**	**0.035**	1.279 (0.968–1.689)	0.083	1.263 (0.945–1.687)	0.115	1.527 (1.175–1.985)	0.002	**1.383 (1.053–1.817)**	**0.020**
**Distress from War Noises**	No	Reference				Reference				Reference			
	Yes	0.650 (0.460–0.918)	0.014	0.870 (0.590–1.282)	0.481	0.813 (0.612–1.081)	0.154	0.900 (0.665–1.218)	0.495	0.755 (0.577–0.987)	0.040	0.863 (0.650–1.147)	0.310
**Changing Place of Living due to War**	No	-	-	-	-	Reference				-	-	-	-
	Yes	-	-	-	-	0.791 (0.584–1.070)	0.128	0.792 (0.576–1.089)	0.151	-	-	-	-
**Model Summary**		Nagelkerke R Square = 0.159; Hosmer and Lemeshow Test (χ^2^ = 6.220; p = 0.623)				Nagelkerke R Square = 0.033; Hosmer and Lemeshow Test (χ^2^ = 10.106; p = 0.258)				Nagelkerke R Square = 0.068; Hosmer and Lemeshow Test (χ^2^ = 13.560; p = 0.094)			

^┼^ OR: Odds Ratio; AOR: Adjusted Odds Ratio; CI: Confidence Interval; Statistically significant values at p<0.05 in the adjusted models are shown in **bold**.

^$^Age is treated as a continuous variable.

## 4. Discussion

Our study sheds the light on an important topic that is considered a threat to global public health, and one of the most common causes of preventable illnesses. During 2002–2003 in Syria, and according to the World Health Organization (WHO) estimates, the overall tobacco smoking was revealed to be around 24.7% (48.0% among males, and 8.9% among females) [23]. Additionally, the prevalence of cigarette smoking was 56.9% among males and 17.0% among females in 2006 [17], while cigarette smoking has reached an overall prevalence of 42.2% in 2014 in Syria [24]. The overall rate of current tobacco smoking (cigarette/shisha) in our study was found to be around 37.9% (53.8% among males, 32.5% among females). Besides, rates of current *cigarette* smoking (exclusive & dual) among males and females were found to be 34.7% and 10.0%, respectively (p<0.001) with an overall rate of 16.4% in the total sample. In comparison with other countries in the EMR, rates of current tobacco smoking had reached 48.7% among males and 29.4% among females in Lebanon, while rates in Jordan were 49.6% among males and 5.7% among females, and in Iraq were 38.2% among males and 1.9% among females [23].

The change in rates of current cigarette smoking (42.2% in 2014 and 16.4% in 2019) could be attributed to the double-edged effects of the war crisis and the associated economic impacts that may decrease the individuals’ accessibility and affordability to tobacco products. This fact was also noticed in the regression model where middle SES has predicted lower odds of being a cigarette smoker, however, this finding was statistically not significant.

In our study, male gender was found to have statistically higher odds of being current cigarette smokers compared to females (AOR = 4.152; 95% CI: 2.842–6.064; p<0.001). This finding is also consistent with most reports that found higher rates of cigarette smoking among males compared to females [17,23,25]. More factors were found to have a significant relationship with current cigarette smoking such as unemployment which predicted lower odds of cigarette smoking, shisha smoking, and overall tobacco use among the participants. This can be explained by the reduced affordability of tobacco products among unemployed individuals due to financial constraints. On the contrary, losing someone due to armed conflicts in Syria has been found to be associated with higher odds of smoking cigarettes. This can be caused by the psychosocial traumas and war-induced stressors that may impact smoking behaviors (increase smoking, initiation of smoking) [14].

Concerning current shisha smoking (exclusive & dual) in our study, the overall rate was estimated to be 29.3% (34.3% among males, 27.6% among females; p = 0.047). In comparison, a study that was conducted in 2003 in Syria, found that shisha was smoked by 25.5% of male university students and 4.9% of female university students [26]. A recent systematic review revealed a high prevalence rate of shisha smoking in the EMR countries among young adults and youths [27], and this raises the alarm about the growing popularity of this tobacco product among the young population, who usually underestimate the health risks of smoking [1]. Moreover, most recent studies that were conducted in Syria reported that the prevalence of shisha smoking was around 20.2% among males and 4.8% among females in 2006 [17], while it was 15.6% among males and 7.4% among females in 2014 [24]. In comparison with other countries in the EMR, shisha smoking was reported among 37.2% of Lebanese youths, reaching up to 65.3% among university students in Lebanon [27]. Other studies also found shisha smoking to be more common in young age groups such as university students and youths as it reached 16.3% in Iran, 32.7% in the West Bank, and 18.9% in Jordan [27]. In the regression models of our study, age was not found to be a statistically significant predictor of cigarette or shisha smoking.

Most people smoke shisha in indoor places where the quality of air can be worsened due to high levels of toxic substances in shisha emissions [28–30]. Shisha is considered as an element of the cultural identity, and it contributes to the sense of togetherness in social events in Syria, while cigarette smoking is usually started in early adolescent life as males in that age group tend to express their identity as “real men” by adopting risky behaviors, which also warns about the cultural influence on tobacco use in the EMR [1]. Interestingly, cigarette smokers feel stigmatized while shisha smoking is perceived as socially acceptable [8]. This also can justify the lower rates of smoking reported previously among females in Syria and the nearby countries. In our study, females were found to be more shisha smokers than cigarette smokers as the rate of current shisha smoking (exclusive & dual) among females was around 27.6% while it was 10.0% for current cigarette smoking (exclusive & dual). This could be explained by the fact that females being able to smoke shisha with friends and social gatherings without being socially stigmatized while cigarette smoking is not considered a social activity, and therefore many females will avoid it as it is not very socially acceptable for women in Syria.

In addition, married participants and those who were unemployed were found to have statistically significant lower odds for shisha smoking married status (AOR = 0.622; 95% CI: 0.402–0.963; p = 0.033, and AOR = 0.679; 95% CI: 0.503–0.916; p = 0.011, respectively). This also can share the same explanation described earlier regarding financial constraints that reversely impact the individuals’ affordability of tobacco products in general. Besides, participants who lost someone due to the war were found to have higher odds of shisha smoking, but this association was found to be statistically not significant in the regression model (model 2).

As described earlier, environmental, and psychosocial stressors may have significant impacts on tobacco use behaviors. For instance, soldiers deployed at wartimes have a higher risk of tobacco smoking [31], and psychologically traumatized individuals (i.e., individuals with PTSD) tend to be more dependent on nicotine [32,33]. In our study, participants who lost someone due to the war were found to have higher odds of overall tobacco smoking as well as cigarette smoking. Unfortunately, and despite tobacco-related harmfulness, tobacco smoking might be adopted by many individuals to cope with stress which can lead to further negative health effects [32–34].

Finally, our current study has many limitations that should be carefully considered when interpreting the findings, and this includes: (i) Using non-probability sampling which limits the generalizability of our findings, (ii) using a web-based survey that affected the representativeness of our sample as most of the participants were under the age of 45 years and from Damascus. This could be attributed to the high level of digital literacy among the young population, with better accessibility to internet services in certain Syrian provinces, (iii) face to face paper-based survey and randomized sampling was not feasible due to administrative issues related to the armed conflicts and safety, (iv) assessment of SES of the participants in our study was based on self-reported states (not validated) as there is no reliable tool to assess SES in Syria, (v) we only assessed current tobacco use regarding cigarettes and shisha as these are the most common forms of tobacco use in the country, however, other types of smoking might be also present, and lastly (vi) the questionnaire represents self-reported states; thus, recall bias could be considered.

## 5. Conclusion

Amid the tobacco epidemic in the region, rates of tobacco use in Syria are still worrying. The Syrian armed conflict and political unrest may possess a double-edged effect on smoking behaviors, in which an increase in tobacco use due to psychosocial and war-induced stressors and/or a decrease in tobacco use due to financial constraints and worsening of the living conditions could be noticed. More stringent and effective implementation of anti-tobacco measures should be advocated in Syria while considering the sociocultural influence on tobacco use among youths and young adults who might underestimate the dangers of tobacco. Tobacco users who adopt smoking to cope with various stressors (e.g., war-induced stressors) should be targeted with well-structured health education, along with appropriate psychological support services.

## Supporting information

S1 File Questionnaire. (DOCX)

## References

[1] Al-Tammemi AB. Tobacco epidemic in Jordan: the time to act is now. Glob Health Promot. 2021. doi: 10.1177/17579759211026181 34269113

[2] Barakat M, Jirjees F, Al-Tammemi AB, Al-Qudah R, Alfoteih Y, Kharaba Z, et al. The Era of E-Cigarettes: A Cross-Sectional Study of Vaping Preferences, Reasons for Use and Withdrawal Symptoms Among Current E-Cigarette Users in the United Arab Emirates. J Community Health. 2021. doi: 10.1007/s10900-021-00967-4 33559828 PMC7871133

[3] Khatatbeh MM, Alkhaldi S, Khader Y, Momani W, Al Omari O, Kheirallah K, et al. Prevalence of nicotine dependence among university students in Jordan: a cross-sectional study. Epidemiol Biostat Public Heal. 2019;16: e13075. doi: 10.2427/13075

[4] National Institute on Drug Abuse. Cigarettes and Other Tobacco Products—Drug Facts. In: [Online] [Internet]. 2021 [cited 1 Aug 2021]. https://www.drugabuse.gov/publications/drugfacts/cigarettes-other-tobacco-products.

[5] Rastam S, Ward KD, Eissenberg T, Maziak W. Estimating the beginning of the waterpipe epidemic in Syria. BMC Public Health. 2004;4: 32. doi: 10.1186/1471-2458-4-32 15294023 PMC514554

[6] Alzyoud S, Weglicki LS, Kheirallah KA, Haddad L, Alhawamdeh KA. Waterpipe Smoking among Middle and High School Jordanian Students: Patterns and Predictors. International Journal of Environmental Research and Public Health. 2013. doi: 10.3390/ijerph10127068 24351734 PMC3881154

[7] Al-Tammemi AB, Barakat M, Al-Tamimi DB, Alhallaq SA, Al Hasan DM, Khasawneh GM, et al. Beliefs Toward Smoking and COVID-19, and The Pandemic Impact on Smoking Behavior and Quit Intention: Findings from a Community-Based Cross-Sectional Study in Jordan. Res Sq. 2021. doi: 10.21203/rs.3.rs-580917/v1 PMC8637701 34866951

[8] Hammal F, Mock J, Ward KD, Eissenberg T, Maziak W. A pleasure among friends: how narghile (waterpipe) smoking differs from cigarette smoking in Syria. Tob Control. 2008;17: e3 LP–e3. doi: 10.1136/tc.2007.020529 18375726

[9] Kassem NOF, Daffa RM, Liles S, Jackson SR, Kassem NO, Younis MA, et al. Children’s exposure to secondhand and thirdhand smoke carcinogens and toxicants in homes of hookah smokers. Nicotine Tob Res. 2014;16: 961–975. doi: 10.1093/ntr/ntu016 24590387 PMC4072898

[10] Kassem NOF, Kassem NO, Jackson SR, Liles S, Daffa RM, Zarth AT, et al. Benzene uptake in Hookah smokers and non-smokers attending Hookah social events: regulatory implications. Cancer Epidemiol biomarkers Prev. 2014;23: 2793–2809. doi: 10.1158/1055-9965.EPI-14-0576 25416714

[11] Kassem NOF, Kassem NO, Liles S, Zarth AT, Jackson SR, Daffa RM, et al. Acrolein Exposure in Hookah Smokers and Non-Smokers Exposed to Hookah Tobacco Secondhand Smoke: Implications for Regulating Hookah Tobacco Products. Nicotine Tob Res. 2018;20: 492–501. doi: 10.1093/ntr/ntx133 28591850 PMC5896480

[12] Idris A, Al Saadi T, Turk T, Alkhatib M, Zakaria M, Sawaf B, et al. Smoking behaviour and patterns among university students during the Syrian crisis. East Mediterr Heal J. 24: 154–160. doi: 10.26719/2018.24.2.154 29748944

[13] Kakaje A, Al Zohbi R, Hosam Aldeen O, Makki L, Alyousbashi A, Alhaffar MBA. Mental disorder and PTSD in Syria during wartime: a nationwide crisis. BMC Psychiatry. 2021;21: 2. doi: 10.1186/s12888-020-03002-3 33388026 PMC7778805

[14] Kheirallah KA, Cobb CO, Alsulaiman JW, Alzoubi A, Hoetger C, Kliewer W, et al. Trauma exposure, mental health and tobacco use among vulnerable Syrian refugee youth in Jordan. J Public Health (Bangkok). 2020;42: e343–e351. doi: 10.1093/pubmed/fdz128 31742341

[15] Kheirallah KA, Veeranki SP, Alzyoud S, Alzoubi A, Walker L, Khader Y, et al. Collision of waterpipe and cigarette smoking epidemics among youth in Arab countries. J Subst Use. 2016;21: 530–536. doi: 10.3109/14659891.2015.1082159

[16] Maziak W, Mzayek F. Characterization of the smoking habit among high school students in Syria. Eur J Epidemiol. 2000;16: 1169–1176. doi: 10.1023/a:1010907724688 11484808

[17] Ward KD, Eissenberg T, Rastam S, Asfar T, Mzayek F, Fouad MF, et al. The tobacco epidemic in Syria. Tob Control. 2006;15 Suppl 1: i24–9. doi: 10.1136/tc.2005.014860 16723671 PMC2563543

[18] Kakaje A, Alhalabi MM, Alyousbashi A, Hamid A, Hosam Aldeen O. Allergic Rhinitis and Its Epidemiological Distribution in Syria: A High Prevalence and Additional Risks in War Time. Biomed Res Int. 2020; 7212037. doi: 10.1155/2020/7212037 32596361 PMC7273446

[19] Kakaje A, Alhalabi MM, Alyousbashi A, Hamid A, Mahmoud Y. Laryngopharyngeal reflux in war-torn Syria and its association with smoking and other risks: an online cross-sectional population study. BMJ Open. 2020;10: e041183. doi: 10.1136/bmjopen-2020-041183 33243809 PMC7692828

[20] World Health Organization. Health Topics—Tobacco Facts. In: [Online] [Internet]. 2021 [cited 2 Feb 2021]. https://www.who.int/health-topics/tobacco#tab=tab_1.

[21] Dean AG, Sullivan KM, Soe MM. OpenEpi: Open Source Epidemiologic Statistics for Public Health. Atlanta, GA, USA; 2013. www.OpenEpi.com.

[22] Irie M, Nakanishi R, Yasuda M, Fujino Y, Hamada K, Hyodo M. Risk factors for short-term outcomes after thoracoscopic lobectomy for lung cancer. Eur Respir J. 2016;48: 495–503. doi: 10.1183/13993003.01939-2015 27174883

[23] World Health Organization. WHO report on the global tobacco epidemic, 2009. In: [Online] [Internet]. 2009 [cited 1 Aug 2021]. https://www.who.int/tobacco/mpower/2009/mpower_report_2009_executive_summary_EN_11b.pdf.

[24] Ward KD, Ahn S, Mzayek F, Al Ali R, Rastam S, Asfar T, et al. The relationship between waterpipe smoking and body weight: population-based findings from Syria. Nicotine Tob Res. 2014/08/05. 2015;17: 34–40. doi: 10.1093/ntr/ntu121 25096252 PMC4351400

[25] Maziak W. Smoking in Syria: profile of a developing Arab country. Int J Tuberc lung Dis Off J Int Union against Tuberc Lung Dis. 2002;6: 183–191. 11934135

[26] Maziak W, Fouad FM, Asfar T, Hammal F, Bachir EM, Rastam S, et al. Prevalence and characteristics of narghile smoking among university students in Syria. Int J Tuberc lung Dis. 2004;8: 882–889. 15260281

[27] Jawad M, Charide R, Waziry R, Darzi A, Ballout RA, Akl EA. The prevalence and trends of waterpipe tobacco smoking: A systematic review. PLoS One. 2018;13: e0192191. doi: 10.1371/journal.pone.0192191 29425207 PMC5806869

[28] Cobb CO, Vansickel AR, Blank MD, Jentink K, Travers MJ, Eissenberg T. Indoor air quality in Virginia waterpipe cafes. Tob Control. 2013;22: 338–343. doi: 10.1136/tobaccocontrol-2011-050350 22447194 PMC3889072

[29] Fiala SC, Morris DS, Pawlak RL. Measuring indoor air quality of hookah lounges. Am J Public Health. 2012/09/20. 2012;102: 2043–2045. doi: 10.2105/AJPH.2012.300751 22994168 PMC3477955

[30] Zhou S, Weitzman M, Vilcassim R, Wilson J, Legrand N, Saunders E, et al. Air quality in New York City hookah bars. Tob Control. 2014/09/16. 2015;24: e193–e198. doi: 10.1136/tobaccocontrol-2014-051763 25232045 PMC4390442

[31] Smith EA, Malone RE. Why strong tobacco control measures “can’t” be implemented in the U.S. Military: a qualitative analysis. Mil Med. 2012;177: 1202–1207. 23113448 10.7205/milmed-d-12-00199 PMC3572716

[32] Feldner MT, Babson KA, Zvolensky MJ. Smoking, traumatic event exposure, and post-traumatic stress: a critical review of the empirical literature. Clin Psychol Rev. 2007;27: 14–45. doi: 10.1016/j.cpr.2006.08.004 17034916 PMC2575106

[33] Greenberg JB, Ameringer KJ, Trujillo MA, Sun P, Sussman S, Brightman M, et al. Associations between posttraumatic stress disorder symptom clusters and cigarette smoking. Psychology of Addictive Behaviors. Leventhal, Adam M.: University of Southern California Keck School of Medicine, 2250 Alcazar Street, CSC 240, Los Angeles, CA, US, 90033, adam.leventhal@usc.edu: American Psychological Association; 2012. pp. 89–98. doi: 10.1037/a0024328 21688875 PMC3307596

[34] Kouvonen A, Kivimäki M, Virtanen M, Pentti J, Vahtera J. Work stress, smoking status, and smoking intensity: an observational study of 46,190 employees. J Epidemiol Community Health. 2005;59: 63–69. doi: 10.1136/jech.2004.019752 15598729 PMC1763376

