# Supplementary material for: Smoking habits and the influence of war on cigarette and shisha smoking in Syria
Source: PLoS One. 2021 Sep 2;16(9):e0256829. doi: 10.1371/journal.pone.0256829 (PMC8412248; doi:10.1371/journal.pone.0256829)
Supplement: S1 File — (DOCX) [file pone.0256829.s001.docx]

**QUESTIONNAIRE ITEMS**

**SECTION ONE: SOCIODEMOGRAPHIC PROFILE**

1. **Age (in years) ………**
2. **Gender:**

- Female
- Male

1. **Marital status:**

- Single (never married, divorced, separated, widowed)
- Married

1. **Educational level:**

- Up to high school
- College/university education

1. **Governorate of current residence:**

- Damascus and Rif Dimashq
- Daraa
- Al-Raqqah
- As-Suwayda
- Deir Ezzor
- Latakia
- Al-Hasakah
- Hama
- Idleb
- Tartous
- Homs
- Aleppo

1. **Employment status:**

- Employed
- Unemployed

1. **Socio-Economic Status (SES):**

- Lower
- Middle
- Upper

1. **Chronic medical conditions**

- Medically free
- Pulmonary conditions (including asthma, allergy)
- Other medical conditions like Hypertension, Diabetes, gastrointestinal, endocrine, rheumatological, neurological…etc

**SECTION TWO: TOBACCO USE PROFILE**

1. **During the past 30 days, have you smoked tobacco products (cigarettes or shisha)?**

- Yes
- No

1. **If answered YES to the previous question, what smoking methods you have smoked specifically?**

- Only cigarettes
- Only Shisha
- Both cigarettes and shisha

1. **In the case of cigarette smoking, please write down for how many years have you been smoking cigarettes? (In years)**
2. **In the case of cigarette smoking, please write down how many packs you smoke in a day? (Every 20 cigarettes correspond to 1 pack)**
3. **In the case of shisha smoking, please write down the average duration you spend in a single shisha session (in hours).**
4. **In the case of shisha smoking, please write down the average number of shisha sessions you smoke in a week.**
5. **In the case of shisha smoking, what is the preferred time for you to smoke shisha?**

- Morning
- In social gatherings
- No specific time

**SECTION THREE: WAR-RELATED IMPACTS**

1. **Did you lose someone due to the Syrian armed conflicts?**

- No
- Yes

1. **Have you changed your usual place of residence due to the Syrian armed conflicts?**

- No
- Yes

1. **Have you been distressed by the war-induced noises? (i.e., bombs and explosions)**

- No
- Yes
